# Low‐Intensity Pulsed Ultrasound Promotes Spinal Cord Injury Recovery by Regulating Microglia‐Mediated Neuroinflammation

**DOI:** 10.1002/cns.70849

**Published:** 2026-03-28

**Authors:** Xiaoyu Li, Yang Liu, Wanghui Liu, Tian Wang, Hanfei Shang, Hongji Zhong, Zixiang Deng, Xiaoyi Zhou, XianZhao Wei

**Affiliations:** ^1^ Department of Orthopedics Shanghai Changhai Hospital Shanghai China

**Keywords:** bioinformatics analysis, inflammation, low‐intensity pulsed ultrasound, microglia, spinal cord injury

## Abstract

**Background:**

Spinal cord injury is a severe central nervous system disorder with high morbidity and mortality, whose pathological process is divided into primary and secondary injury. The regulation of neuroinflammation is a critical link in secondary injury, forming a positive feedback loop involving microglia and peripheral immune cells. Low‐intensity pulsed ultrasound, a noninvasive physical therapy, exhibits significant potential in regulating inflammation and promoting microangiogenesis in orthopedics and nerve repair, providing new insights for intervening in the secondary pathological cascade of SCI. However, its therapeutic effects and underlying molecular mechanisms in SCI treatment have not been systematically and in‐depth explored.

**Methods:**

A total of 88 Sprague–Dawley rats were randomly divided into the sham operation group, SCI group, and SCI + LIPUS treatment group (nine subgroups with different parameter combinations) to establish a T10 segment SCI model. The SCI + LIPUS group received LIPUS intervention (20 min/day, 5 days/week) starting 1 day after modeling. Basso, Beattie, and Bresnahan locomotor scores were used to evaluate hindlimb motor function at multiple time points postoperatively. On Day 28, spinal cord samples were collected for hematoxylin–eosin staining and immunofluorescence staining to assess cavity area, microglial infiltration, and inflammatory microenvironment changes. In in vitro experiments, a lipopolysaccharide‐induced BV2 microglial polarization model was established, followed by LIPUS intervention. Cell Counting Kit‐8 assay, quantitative polymerase chain reaction, immunofluorescence staining, and Transwell co‐culture with HT22 neurons combined with Western blot were used to detect cell viability, inflammatory factor expression, and neuronal apoptosis. RNA sequencing and bioinformatics analysis (GO/KEGG enrichment, protein–protein interaction network construction) were conducted on spinal cord tissues from the SCI and SCI + LIPUS groups to screen differentially expressed genes and hub genes.

**Results:**

BBB scores showed that the SCI + LIPUS group (especially with the parameters of 180 mW/cm^2^ and 1.5 MHz) exhibited significantly better hindlimb motor function than the SCI group from day 7 postoperatively, persisting until day 28 (*p* < 0.05). HE and immunofluorescence staining indicated that LIPUS reduced spinal cord cavity area, inhibited microglial activation, promoted M2‐type microglial polarization, and alleviated secondary inflammation. In vitro experiments confirmed that LIPUS enhanced BV2 cell viability and proliferation, upregulated the expression of anti‐inflammatory factors (IL‐10, Arg‐1), downregulated pro‐inflammatory factors (IL‐6, TNF‐α), and suppressed HT22 neuron apoptosis by inhibiting inflammatory factor secretion from BV2 cells. RNA sequencing identified 67 DEGs (26 upregulated, 41 downregulated) between the SCI + LIPUS and SCI groups, enriched in immune defense, extracellular matrix remodeling, NOD‐like receptor, and IL‐17 signaling pathways. Hub genes including CXCR2 were identified, and CXCR2 downregulation was confirmed to be involved in LIPUS‐mediated anti‐inflammatory effects.

**Conclusion:**

LIPUS effectively alleviates neuroinflammation after SCI and promotes neurological function recovery by regulating microglia‐mediated neuroinflammation, whose mechanism is associated with the modulation of NOD‐like receptor, IL‐17 signaling pathways and the downregulation of hub genes such as CXCR2. This study provides a scientific basis for the clinical application of LIPUS in SCI treatment and a theoretical foundation for exploring multi‐modal therapeutic strategies combining LIPUS with other therapies.

## Graphical abstract

Low‐intensity pulsed ultrasound (LIPUS) exerts a neuroprotective effect in spinal cord injury (SCI) by regulating microglial polarization: it inhibits pro‐inflammatory M1 phenotype activation, promotes anti‐inflammatory M2 phenotype polarization, alleviates neuroinflammation, inhibits neuronal apoptosis, reduces spinal cord cavity formation, and enhances neuronal survival, thus providing a noninvasive therapeutic strategy for SCI.

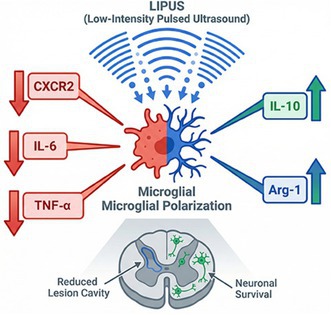

## 1 Introduction

Spinal cord injury (SCI) is a severe disorder of the central nervous system, with a global incidence of approximately 105 cases per million population [1] and an even higher rate of 138.7 cases per million population in China [2], showing a continuous upward trend in recent years. Common etiologies include traffic accidents [3], violent trauma, accidental falls [4], spinal infections [5], ischemia/reperfusion injury, and tumors [6]. SCI not only causes complete or partial loss of sensory [7], motor, and autonomic functions below the injury level but also leads to severe complications such as bedsores, urinary tract infections [8], chronic pain, spasticity, as well as mental health issues [9] including depression [10] and anxiety [11], imposing a heavy burden on patients and their families. The annual treatment cost for patients with severe tetraplegia even exceeds one million dollars [12], with immeasurable losses in social productivity [13, 14].

The field of SCI treatment faces numerous challenges. On one hand, the capacity for neural regeneration after SCI is extremely limited [15], on the other hand, complex secondary injury processes such as inflammatory responses [16] and apoptosis [17] further exacerbate neural damage. Although early surgical decompression, drug therapy, and rehabilitation training can alleviate symptoms to some extent [18, 19] and promote partial neurological recovery, achieving comprehensive repair of advanced neurological functions remains elusive [20, 21]. Emerging technologies such as stem cell therapy [22], tissue engineering, and biological scaffolds [23] have shown great potential, yet they face insurmountable technical bottlenecks in achieving effective neural regeneration and establishing functional neural connections, along with issues such as immune rejection, potential side effects, ethical controversies, and high costs, restricting their widespread clinical application.

Pathologically, SCI is categorized into primary and secondary injury [24]. Primary injury, caused by mechanical damage, is irreversible. Secondary injury, triggered by primary injury, involves pathological processes including vascular damage [25], inflammatory response [26], oxidative stress [27], apoptosis [28], and glial scar formation [29], which are reversible. Among these, neuroinflammation, mediated by microglia [30], astrocytes [31], and neutrophils [32], is a critical pathological process in secondary injury, playing a pivotal role in restoring tissue homeostasis and achieving optimal recovery after SCI. As the primary immune cells and resident macrophages in the central nervous system, microglia are crucial for maintaining CNS homeostasis and regulating immune responses. Under physiological conditions, microglia clear metabolic waste, pathogens, and apoptotic cells through phagocytosis to maintain internal environmental stability. Under pathological conditions such as SCI, microglia are rapidly activated, migrate to the injury site, and release inflammatory factors to participate in immune defense; however, excessive activation exacerbates neural damage. Microglia exhibit two phenotypes: M1 (pro‐inflammatory, involved in immune defense [33]) and M2 (anti‐inflammatory, promoting neural repair [34]). The phenotypic shift has become a key target in SCI treatment research.

Low‐intensity pulsed ultrasound (LIPUS), a noninvasive physical therapy, exerts biological effects through low‐intensity pulsed waves, generating mechanical stimulation, mild thermal effects, and regulating the cellular and tissue microenvironment. It has been reported to promote tissue repair and neural regeneration. Extensive studies have demonstrated its efficacy in accelerating fracture healing [35], promoting tendon and cartilage regeneration [36], and treating traumatic brain injury [37]. However, research on its application in SCI treatment is still in its infancy, with a lack of systematic in‐depth exploration of its therapeutic efficacy and underlying molecular mechanisms, and no clear identification of its specific molecular targets in SCI repair.

In this study, we innovatively conduct in‐depth research on the application of LIPUS in SCI treatment, and the research has multiple innovative points that fill the gaps in the existing field. First, we bridge the gap between the mature orthopedic application of LIPUS and the emerging research of SCI repair by combining in vivo rat T10 segment SCI model experiments and in vitro BV2 microglia‐HT22 neuron co‐culture experiments, systematically and comprehensively verifying the therapeutic efficacy of LIPUS in SCI treatment for the first time in a multi‐dimensional way. Second, we integrate LIPUS intervention with transcriptome sequencing and bioinformatics analysis for the first time in the field of LIPUS and SCI research, screening out differentially expressed genes related to LIPUS‐regulated SCI repair and identifying key signaling pathways and hub genes involved in the repair process, which digs out the specific molecular targets of LIPUS in SCI treatment that have not been reported in previous orthopedic studies of LIPUS. Third, we construct a complete “phenomenon‐mechanism‐target” research chain for LIPUS in SCI repair: on the basis of confirming the phenomenon that LIPUS regulates microglial polarization to alleviate neuroinflammation, we use a variety of technical means to clarify its underlying molecular mechanism, and screen out potential target genes for regulating microglial polarization, breaking the limitation of only phenomenological observation in previous related researches. Fourth, we optimize and unify the LIPUS parameter system for SCI treatment, confirming the optimal parameter combination for both in vivo animal and in vitro cell experiments, ensuring the consistency of biological effects at cellular and tissue levels, and providing a standardized parameter reference for subsequent related research and clinical transformation.

This study conducted in vitro interventions on BV2 cells and in vivo experiments using a Sprague–Dawley male rat SCI model to determine the effects of LIPUS on BV2 cells and its role in promoting SCI recovery in rats. Transcriptome analysis was used to reveal the potential molecular mechanisms of LIPUS in SCI repair, exploring its regulatory effects on microglial activity and immune modulation. The research results are expected to provide a new scientific basis and technical route for the clinical application of LIPUS in SCI treatment and lay a foundation for the development of multi‐modal therapeutic strategies combining LIPUS with other therapies.

## 2 Materials and Methods

### 2.1 Animals and Materials

A total of 88 male Sprague–Dawley rats (8 weeks old, 200 g) were used for in vivo experiments, provided by Shanghai Super‐B&K Laboratory Animal Co. Ltd. BV2 cells used for in vitro experiments were purchased from the Cell Bank of the Chinese Academy of Sciences (Shanghai, China).

The rats were randomly divided into 11 groups with 8 rats per group: 1 sham operation (Sham) group, 1 spinal cord injury (SCI) group, and 9 treatment (SCI + LIPUS) groups (assigned to different intensity and frequency combinations). To ensure no baseline differences among groups that could affect experimental outcomes, baseline data of all rats were collected 1 day before surgery (pre‐modeling). The measured indicators included: (1) Body weight: measured using an electronic balance (accuracy: 0.1 g), with a mean ± standard deviation ($\bar{x}$ ± *s*) of 200.3 ± 8.5 g in the Sham group, 201.1 ± 7.9 g in the SCI group, and 199.8 ± 8.2 to 202.5 ± 7.7 g in the 9 SCI + LIPUS subgroups; (2) Baseline Basso, Beattie, and Bresnahan (BBB) locomotor score: all rats exhibited normal hindlimb motor function with a score of 21 points (full score); (3) General health status: no rats showed abnormalities such as abnormal gait, loss of appetite, or signs of infection. Statistical analysis (one‐way ANOVA) revealed no significant differences in body weight among all groups (*p* > 0.05), confirming that the grouping was balanced and baseline characteristics were comparable. The SCI group was subjected to spinal cord clamping to induce injury. Similarly, the SCI + LIPUS groups underwent SCI modeling but received LIPUS treatment after injury. In contrast, the sham operation group underwent the same surgical procedure without spinal cord clamping, serving as the control group. During the experiment, two rats were excluded due to unsuccessful modeling (failure to meet inclusion criteria), resulting in a final valid sample size of 86 rats (attrition rate 2.3%). Animals were housed in a Specific Pathogen Free (SPF) facility under controlled environmental conditions: temperature 24°C ± 2°C, relative humidity 40%–70%, and a standard 12‐h light/dark cycle. They had ad libitum access to food and water.

### 2.2 Surgical Procedures

Rats were anesthetized with 5% isoflurane and placed in the prone position. Anesthesia was maintained during surgery using a gas anesthetic machine with isoflurane (0.5%–2%) and oxygen (0.5–0.8 L/min). The surgical area was shaved and disinfected with povidone‐iodine solution. A 3 cm midline dorsal incision was made, and paravertebral muscles were bluntly dissected to expose the thoracic vertebrae. A laminectomy was performed at the T10 level using rongeurs to fully expose the spinal cord. The spinal cord was compressed with a 70 g aneurysm clip (Aesculap, Tuttlingen, Baden‐Württemberg, Germany) for 30 s. Successful modeling was indicated by tail spasm, bilateral hindlimb retraction, flapping and paralysis, and dural congestion and edema. Rats that failed to exhibit these signs were excluded from the study (*n* = 2). Hemostasis was performed, and the skin and fascia were sutured. Antibiotics were intramuscularly injected twice daily for 3 days to prevent infection. To prevent urinary retention and infection, manual bladder expression was performed daily at 08:00, 14:00, and 20:00. Each session lasted approximately 30–60 s until the bladder was fully emptied. Surgeries were performed by X.L., Y.L., W.L. and T.W.

### 2.3 LIPUS Treatment

**FIGURE 1:**
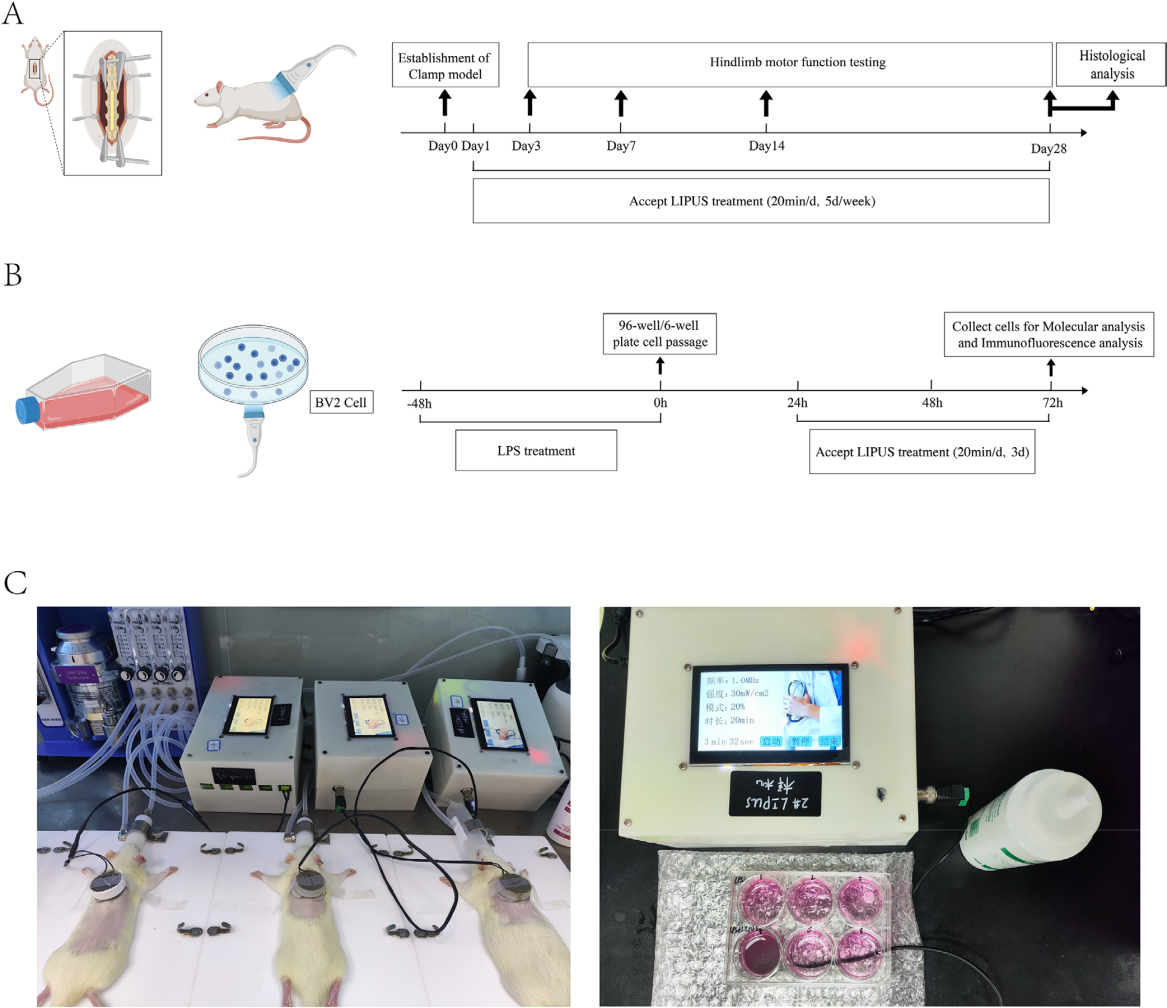
Experimental design for both the in vivo and in vitro studies. (A) Timeline of the treatment of SCI in rats. The histological evaluation included HE staining and immunofluorescence. (B) Timeline of the induction and treatment for BV2 cells. Molecular analysis mainly refers to qPCR. (C) In vivo and in vitro experimental images.

The timeline of in vivo SCI model treatment, including LIPUS intervention and motor function testing, is shown in Figure 1A. A self‐developed multi‐parameter adjustable low‐intensity pulsed ultrasound therapy device was used in this experiment, co‐designed by the First Affiliated Hospital of Naval Medical University and the University of Shanghai for Science and Technology. It adopted a modular design, comprising a power module, micro‐control unit, high‐frequency sine wave signal generator, programmable amplifier, power amplifier, and piezoelectric transducer impedance matching circuit. After assembly, rigorous safety tests and system debugging were conducted to ensure safe and effective in vitro intervention, and the probe was verified to stably output ultrasound signals with preset frequency and intensity using an oscilloscope. The SCI + LIPUS group received LIPUS treatment starting 1 day after surgery (20 min per day, 5 days per week). Different frequencies and intensities were adjusted to output various parameter combinations, with a fixed duty cycle of 20%, resulting in nine parameter combinations (Table 1). The nine treatment (SCI + LIPUS) groups were intervened with different LIPUS parameter combinations. Rats were maintained under isoflurane anesthesia during LIPUS treatment. As shown in Figure 1C, the LIPUS probe was carefully fixed on the skin above the fusion site, and coupling gel was applied to ensure contact between the probe and the skin. LIPUS treatments were performed by X.L., Y.L., W.L., T.W., H.S.

**TABLE 1:** Measurement of ultrasound parameters of the LIPUS system.

Frequency (MHz)	Intensity (mW/cm^2^)		
	30	90	180
1.0	Combination 1	Combination 2	Combination 3
1.5	Combination 4	Combination 5	Combination 6
2.0	Combination 7	Combination 8	Combination 9

*Note:* The duty cycle was set to 20% for all groups.

### 2.4 Basso, Beattie, and Bresnahan (BBB) Scoring

The BBB locomotor rating scale was used to evaluate the recovery of hindlimb motor function in rats. Double‐blind scoring was performed on Days 1, 3, 5, 7, 14, 21, and 28 postoperatively. Prior to the experiment, all evaluators underwent standardized training to unify the scoring criteria, ensuring an inter‐rater reliability of > 95%. During scoring, rats were placed individually in a specially designed arena for free movement. Hindlimb activities were carefully observed and video‐recorded. Two independent researchers assessed the BBB score while blinded to the experimental grouping to minimize subjective bias. In cases of significant discrepancies (> 1 point), the video was reviewed, and a consensus was reached through discussion or arbitration by a senior researcher. All rats followed a unified scoring protocol at each assessment time point to ensure data comparability and experimental rigor. BBB scoring was performed by X.L. and Y.L.

### 2.5 Histological Analysis

Spinal cord samples from each group were subjected to hematoxylin–eosin (HE) staining. After the final LIPUS treatment, rats were perfused with normal saline, followed by 4% paraformaldehyde. Subsequently, 1 cm spinal cord segments centered at the injury site were collected from the SCI and SCI + LIPUS groups, and corresponding sections from the sham operation group. Spinal cord tissues were fixed in 4% paraformaldehyde for 48 h, dehydrated, embedded in paraffin, sectioned, stained with HE, and mounted with mounting medium. Stained tissue sections were observed and photographed using a light microscope to evaluate morphological characteristics, cellular changes, and structural integrity.

### 2.6 Quantitative Image Analysis Protocol

ImageJ software (National Institutes of Health, Bethesda, MD, USA) was used to measure the size of cavities within the spinal cord. Digital images of stained sections were captured under a light microscope equipped with a high‐resolution digital camera at a fixed magnification. For quantitative analysis, three discontinuous sections spanning the lesion epicenter were selected for each animal. Cavity areas were manually outlined using the software's drawing tool by an investigator blinded to the grouping, and the area within the defined boundaries was automatically calculated. The percentage of cavity size was then computed as: Cavity size (%) = (Injury area/Total spinal cord area) × 100. The values from the three sections were averaged to represent the cavity size for each rat. To ensure the accuracy and objectivity of quantitative analysis, the following details were strictly implemented: (1) Number of fields of view: For each of the three discontinuous sections per rat, three nonoverlapping fields of view centered on the lesion epicenter were randomly selected (total 9 fields of view per rat), avoiding overlapping regions to eliminate duplicate data collection. (2) Randomness of field selection: A random number table was used to determine the coordinates of the fields of view, ensuring no subjective bias in selection. (3) Exclusion criteria for slices: Sections with blurred edges, uneven staining, tissue damage, or incomplete spinal cord structure were excluded from analysis; only slices with clear tissue boundaries, uniform staining, and intact lesion regions were included to minimize quantitative errors. All image acquisition and analysis were performed by an investigator blinded to the experimental groups to further reduce systematic bias.

### 2.7 Immunofluorescence Staining

Following the same steps as HE staining, spinal cord tissues were removed after cardiac perfusion, fixed in 4% paraformaldehyde, dehydrated, paraffin‐embedded, sectioned, and dewaxed. Fixed sections were incubated with ionized calcium‐binding adapter molecule 1 (IBA1) antibody, and anti‐fluorescence quenching mounting medium was used to preserve fluorescence signal stability. Sections were then observed and photographed using a fluorescence microscope.

### 2.8 Cell Culture

BV2 cells were cultured in Dulbecco's Modified Eagle's Medium (DMEM; Thermo Fisher Scientific, Wilmington, USA) supplemented with 10% fetal bovine serum (FBS; Gibco, Australia) and 100 U/mL penicillin–streptomycin, and maintained in a 37°C incubator with 5% CO_2_.

### 2.9 Microglial Polarization

BV2 cells were divided into three groups: control group, lipopolysaccharide (LPS) group, and LPS + LIPUS group. Based on our pilot experiments and previous studies [38, 39], a concentration of 1 μg/mL was selected to ensure effective M1 polarization while maintaining cell viability (> 85%). Therefore, BV2 cells in the LPS and LPS + LIPUS groups were cultured in polarization medium containing 10% FBS and 1 μg/mL LPS for 48 h. The LPS + LIPUS group concurrently received LIPUS treatment (180 mW/cm^2^, 1.5 MHz, 20 min daily) during this period. Cells in the control group received no treatment. After the 48‐h polarization period, cells from all three groups were harvested and re‐seeded into 96‐well and 6‐well plates for subsequent viability assays and molecular analysis.

### 2.10 LIPUS Treatment of Cells

The timeline of in vitro BV2 cell experiments is shown in Figure 1B. BV2 cells were cultured in 96‐well and 6‐well plates for 1 day to allow adhesion, followed by LIPUS treatment (20 min per day for 3 days). LIPUS device parameters were as follows: intensity 180 mW/cm^2^, ultrasound frequency 1.5% ± 5% MHz, duty cycle 20%. The LIPUS probe was carefully fixed to the bottom of the plate using coupling gel to ensure contact (Figure 1C).

### 2.11 Cell Counting Kit‐8 (CCK‐8) Viability Assay

The CCK‐8 assay (UElandy, Suzhou, Jiangsu, China) was used to evaluate the proliferative activity of BV2 cells after LIPUS treatment. At 72 h after LIPUS treatment, BV2 cell status was observed under a microscope. The medium was then replaced with CCK‐8 reagent (10 μL CCK‐8 reagent + 90 μL complete medium) and incubated in a 37°C, 5% CO_2_ incubator for 2 h in the dark. After incubation, absorbance at 450 nm was measured using a multifunctional microplate reader.

### 2.12 Quantitative Polymerase Chain Reaction (qPCR)

Total RNA was isolated from cells using TRIzol reagent (Beyotime, Shanghai, China) according to the manufacturer's instructions. RNA concentration and purity were measured using an EPOCH2 NSC‐SN (Agilent Technologies, Santa Clara, California, USA). RNA was reverse‐transcribed into complementary DNA (cDNA) using PrimeScript RT MasterMix (Takara Bio, Shiga, Japan). PCR was performed using Hieff qPCR SYBR Green MasterMix (Yeasen Bio, Shanghai, China) and primers on an Applied Biosystems QuantStudio 3 (Thermo Fisher Scientific, Massachusetts, USA). Primer sequences used in this study are listed in Table 1. Each sample was analyzed in triplicate to verify yield. Relative gene expression levels were determined using the 2^−ΔΔCt^ method. In this study, β‐actin was selected as the internal reference gene, and its expression stability was validated.

### 2.13 Cellular Immunofluorescence

Bv2 cell climbing slices were prepared. After LIPUS treatment, cells were washed three times with phosphate‐buffered saline (PBS), fixed with 4% paraformaldehyde for 15 min, and washed again. Cells were permeabilized with 0.3% Triton X‐100 at room temperature for 25 min, blocked with 1X animal‐free blocking solution for 15 min, and incubated with primary antibodies (rabbit anti‐IBA1 monoclonal antibody 1:200, rabbit anti‐Arg‐1 monoclonal antibody 1:200, and rabbit anti‐iNOS monoclonal antibody 1:200) at 4°C overnight. The next day, cells were washed three times with PBS, incubated with horseradish peroxidase‐conjugated goat anti‐rabbit IgG (1:50) at room temperature for 1 h, followed by incubation with Cy3‐conjugated secondary antibody for 15 min. After washing, slides were mounted in medium containing 4′,6‐diamidino‐2‐phenylindole (DAPI). Fluorescence was detected using a fluorescence microscope, and the average signal intensity of IBA1, iNOS, and Arg‐1 immunofluorescence was quantified using ImageJ software (Media Cybernetics, Maryland, USA).

### 2.14 HT22 Cell Transwell Co‐Culture

HT22 neuronal cells were expanded and seeded into the lower chamber of 24‐well plates at a density of 1 × 10^5^ cells/well, cultured for 24 h to ensure adhesion and adaptation. Transwell inserts with appropriate size (6.5 mm diameter, 0.4 μm pore size semipermeable membrane) were used to achieve noncontact co‐culture. BV2 cells from the Sham, LPS, and LPS + LIPUS groups were seeded onto the upper membrane of Transwell inserts at approximately 1 × 10^5^ cells/insert in 100 μL DMEM complete medium. The seeding ratio of BV2 cells (upper chamber) to HT22 cells (lower chamber) was 1:1. This ratio was selected based on classic microglia–neuron co‐culture protocols in the field and verified by pilot experiments, ensuring that inflammatory factors secreted by BV2 cells can effectively act on HT22 neurons while avoiding microenvironment interference caused by excessive cell crowding. Inserts with BV2 cells were gently placed into 24‐well plates containing HT22 cells, allowing indirect contact between BV2 cells in the upper chamber and HT22 cells in the lower chamber via the semipermeable membrane. The entire co‐culture system was incubated in a 37°C, 5% CO_2_ incubator for 24 h, enabling cytokines or metabolites secreted by BV2 cells in the upper chamber to act on HT22 cells in the lower chamber through the membrane. After co‐culture, supernatants were collected for ELISA to detect inflammatory factor expression, and HT22 cells in the lower chamber were collected for Western blot to measure protein level changes, evaluating the effects of different treatments on HT22 cell function and status.

### 2.15 Sequencing Sample Preparation

After LIPUS treatment, rats were sacrificed, and spinal cord tissues at the injury site were collected from the SCI and SCI + LIPUS groups. Total RNA was extracted using TRIzol reagent (Invitrogen). Isolated RNA was further purified using an RNA Clean XP Kit to remove residual DNA and other impurities. RNA concentration (> 50 ng/μL) and purity (OD260/280 > 1.8) were detected using a NanoDrop ND‐2000 spectrophotometer (Thermo Fisher Scientific) to ensure sample quality for sequencing. RNA integrity (RIN value) was further assessed using an Agilent 2100 Bioanalyzer (Agilent Technologies). mRNA was specifically captured from total RNA using VAHTS mRNA Capture Beads (Vazyme). Captured mRNA was fragmented and reverse‐transcribed into cDNA using the VAHTS Universal V6 RNA‐seq Library Prep Kit (Vazyme).

### 2.16 Transcriptome Sequencing

Each group (SCI and SCI + LIPUS) included 3 biological replicates to ensure the reliability of differential gene screening. cDNA libraries were purified and size‐selected using VAHTS DNA Clean Beads (Vazyme). Purified libraries were quality‐controlled using the ExKubit dsDNA HS Assay Kit (Excell) and Agilent D1000 Reagents (Agilent) to ensure library fragment size and concentration met sequencing requirements. Paired‐end sequencing (PE150 mode) was performed on an Illumina NovaSeq 6000 platform.

### 2.17 Bioinformatics Analysis

Screening of differentially expressed genes (DEGs): DEGs under LIPUS treatment were identified with criteria of |log2 fold change| ≥ 1 and *p* < 0.05.

Functional enrichment analysis: Gene Ontology (GO) functional enrichment analysis and Kyoto Encyclopedia of Genes and Genomes (KEGG) pathway enrichment analysis were performed on DEGs to reveal significantly enriched biological functions and signaling pathways involved in LIPUS treatment. GO analysis annotated DEGs from three dimensions: biological process (BP), cellular component (CC), and molecular function (MF). KEGG pathway enrichment analysis focused on key signaling pathways involving DEGs, providing insights into the molecular mechanisms of LIPUS.

### 2.18 Statistical Analysis

Data are presented as mean ± standard deviation (*X* ± *S*). One‐way analysis of variance (ANOVA) was used to test overall differences, followed by Tukey's multiple comparison test for pairwise comparisons. A *p*‐value < 0.05 was considered statistically significant. All data analyses were performed using SPSS 22.0 (IBM, New York, USA), and all statistical graphs were generated using GraphPad Prism 10 (GraphPad Software, California, USA).

## 3 Results

### 3.1 BBB Scores

**FIGURE 2:**
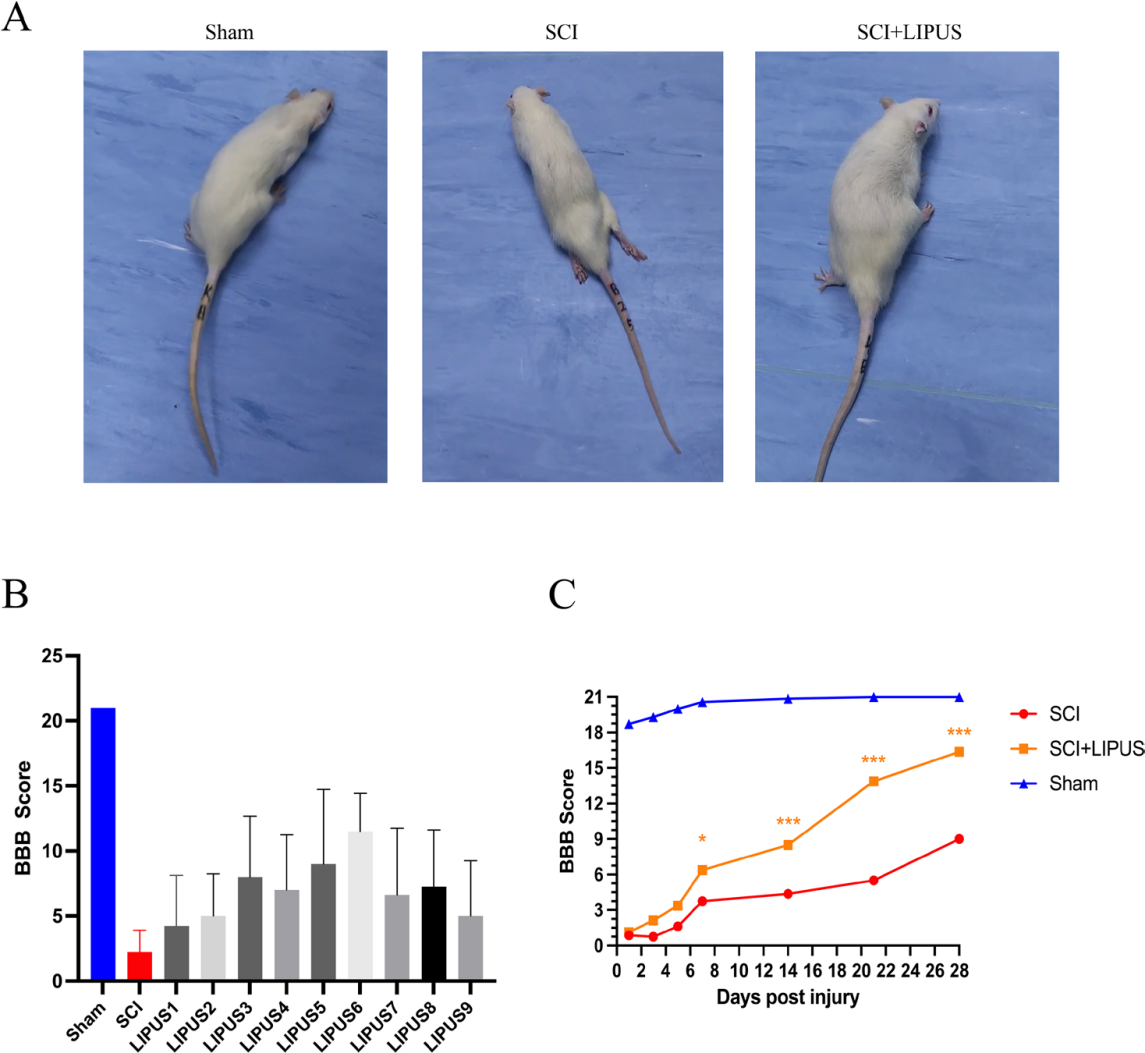
LIPUS promotes locomotor recovery. (A) Representative images of BBB test in different groups. (B) The Basso Beattie Bresnahan (BBB) locomotion scores for motor function evaluation under different parameter combinations at 28 days. (C) Results of BBB score for parameter combination 6 (*n* = 8, compared with the SCI group; group; *p < 0.05, **p < 0.01, ***p < 0.001; *n* = 8 in each group).

After SCI modeling, all groups except the Sham group scored no more than 3 points, indicating successful modeling. Figure 2A shows significant differences in hindlimb motor function among groups. As shown in Figure 2B, among all treatment groups, Combination 6 exhibited the most significant therapeutic effect, achieving the best outcome. Rats in the sham operation group maintained normal hindlimb posture and walking ability, while the SCI group showed severe motor dysfunction, including obvious dragging gait and loss of weight‐bearing capacity, reflecting the severe impact of SCI on the central nervous system. Results of SCI rats intervened with Parameter Combination 6 are shown in Figure 2C: the SCI + LIPUS group showed significant improvement starting from Day 7 postoperatively, persisting until Day 28, with significantly higher scores than the SCI group (*p* < 0.05), indicating that LIPUS treatment effectively promoted neurological function recovery after SCI.

### 3.2 Histological Analysis

**FIGURE 3:**
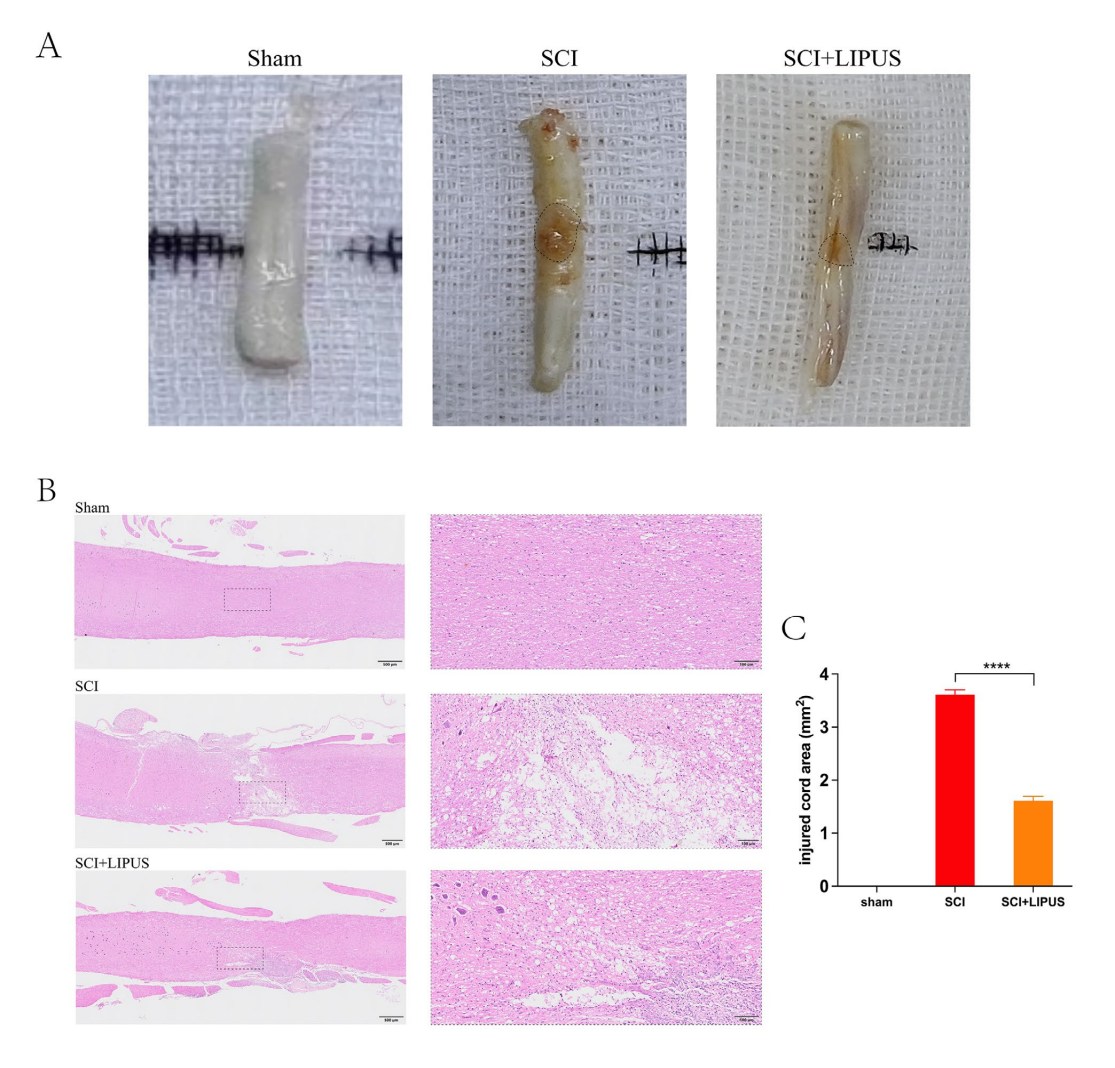
LIPUS improves pathology and morphology of the spinal cord after injury. (A) Gross morphology of the injured spinal cords in Sham SCI and SCI + LIPUS rat at Day 28 post‐SCI. (B) HE staining of the injured center of spinal cord at 28 days post‐injury. Scale bar = 200 μm. (C) Quantification of area of the injured core in the spinal cord at 28 days post‐injury. ****p < 0.0001, *n* = 10 in each group.

Gross morphological examination of the spinal cord revealed significant differences among groups. As shown in Figure 3A, the sham operation group showed intact spinal cord tissue without any signs of injury. In contrast, the SCI group exhibited obvious edema at the injury site, reflecting severe tissue damage. In the SCI + LIPUS group, edema was significantly reduced, indicating that LIPUS treatment contributed to spinal cord repair and alleviated tissue damage after SCI. HE staining further supported these findings, showing enhanced tissue repair and regeneration in the SCI + LIPUS group. The sham operation group maintained normal spinal cord structure without signs of inflammatory cell infiltration. Conversely, the SCI group showed tissue architecture disruption, large cavity formation, and extensive inflammatory cell infiltration. In the SCI + LIPUS group, cavity size was reduced, tissue arrangement was improved, and inflammatory cell infiltration was significantly decreased (Figure 3B). Figure 3C further quantified the reduction in the percentage of injury area in the LIPUS group: cavity area was significantly increased in the SCI group compared to the sham operation group (no cavity formation), while cavity area was significantly reduced in the SCI + LIPUS group, indicating that LIPUS treatment was effective in reducing injury area after SCI. These results demonstrate that LIPUS treatment effectively reduces tissue damage and promotes spinal cord repair after SCI.

### 3.3 Immunofluorescence Staining

**FIGURE 4:**
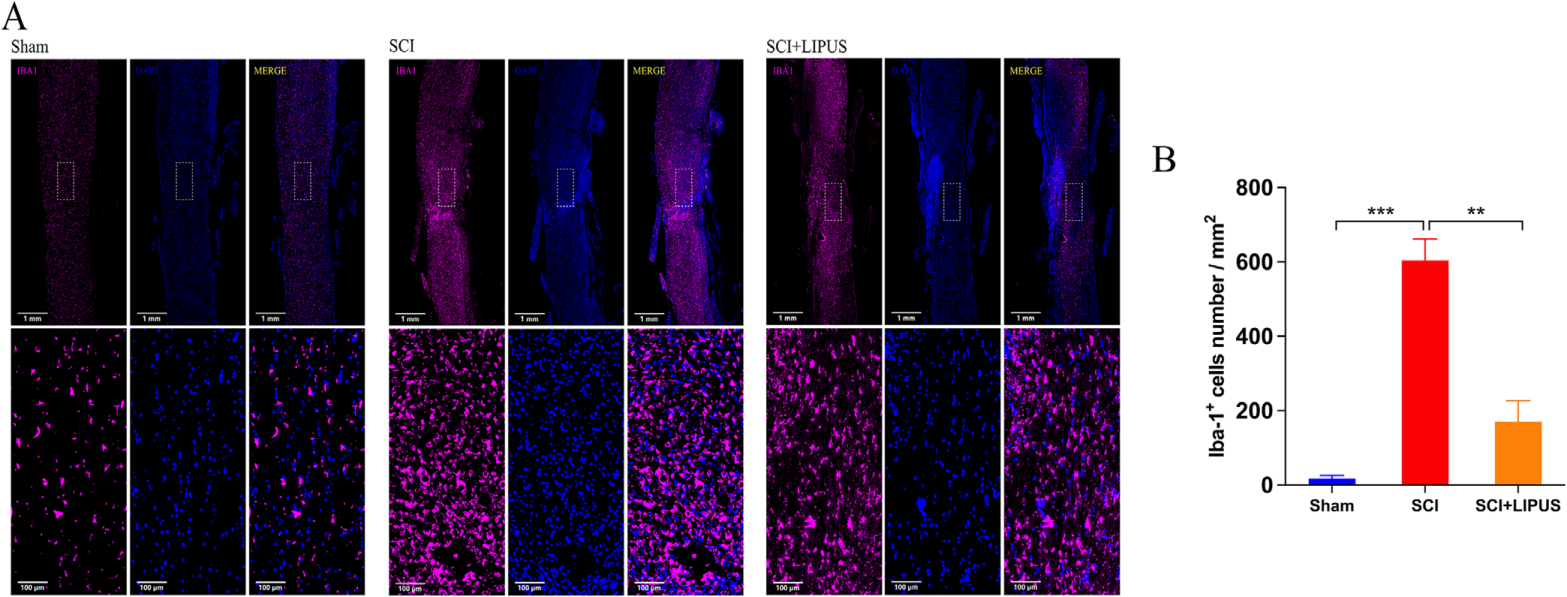
LIPUS alleviates glial accumulation and promotes neural repair. (A) Immunofluorescence shows the distribution of IBAl (pink), and nuclei (blue) in the lesion site of spinal cord at 28 days post‐injury. Scale bar = 1 mm and 100 μm. (B) Quantification of the IBAl‐positive area in the spinal cord at 28 days post‐SCI. **p < 0.01, ***p < 0.001; *n* = 10 in each group.

Longitudinal sections of spinal cord tissue at 28 days post‐SCI were subjected to IBA1 immunofluorescence staining to observe microglial activation after LIPUS treatment. As shown in Figure 4A, consistent with HE staining results, LIPUS treatment significantly reduced the infiltration of microglia (IBA1^+^) at 28 days post‐injury. High‐magnification observation revealed that spinal cord tissue in the SCI group was filled with a large number of pink‐labeled reactive microglia, with obvious inflammatory infiltration, while IBA1 immunofluorescence intensity was significantly lower in the SCI + LIPUS group than in the SCI group (Figure 4B, *p* < 0.01). These results indicate that LIPUS can alleviate inflammatory responses at the injury site by reducing the number or activity of microglia, creating a more favorable microenvironment for neural regeneration.

### 3.4 CCK‐8 Viability Assay

**FIGURE 5:**
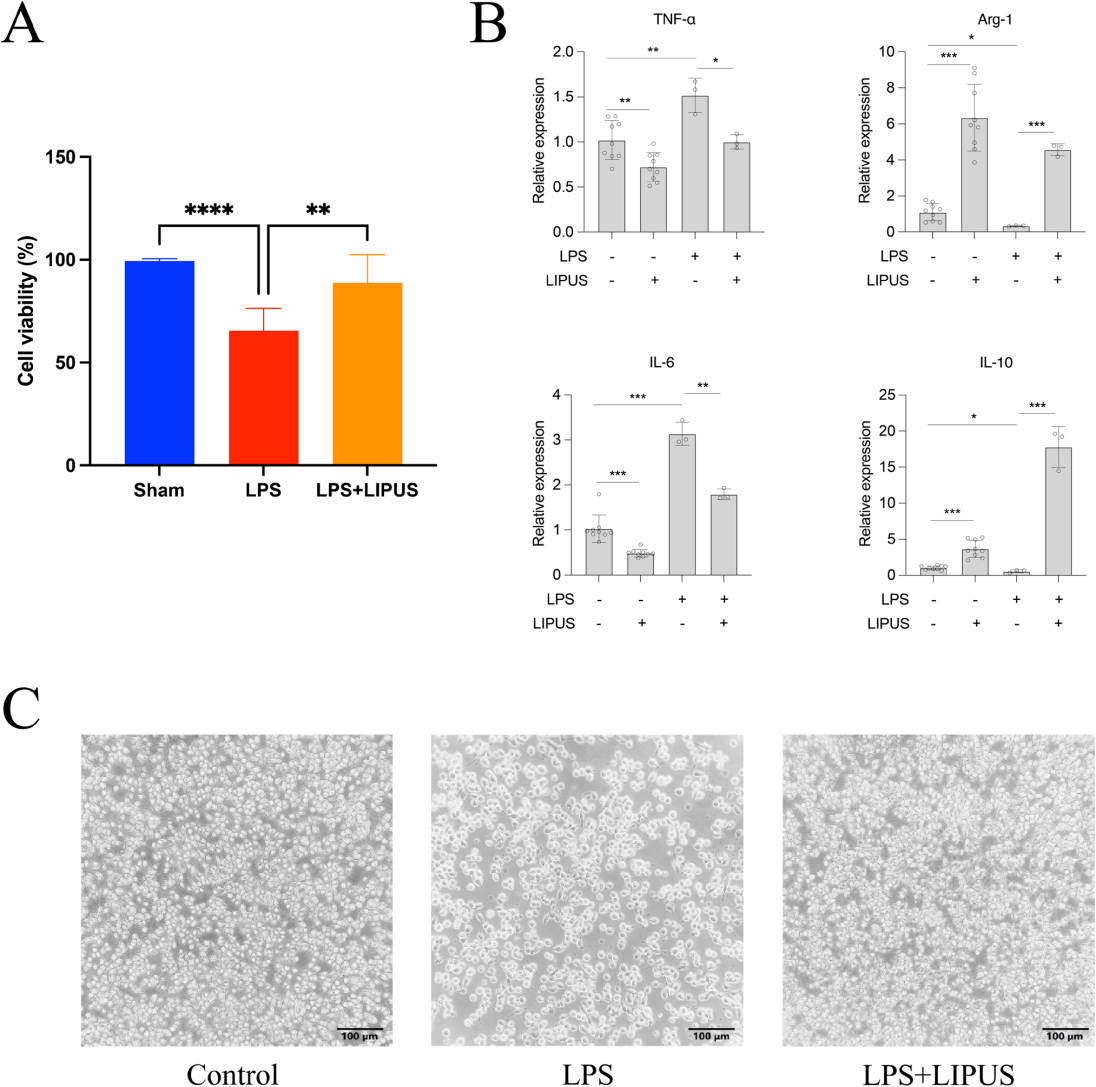
Effects of LIPUS treatment on the microglial cells. (A) Cellular proliferation activity assessment after LIPUS stimulation. (B) Cells were evaluated for TNF‐α, Arg‐1, IL‐6 and IL‐10 mRNA expression at 72 h after LIPUS treatment. (C) Morphological observation of microglia. (Scale bar 100 μm).

As shown in Figure 5C, cells in the control group maintained normal and dense distribution. In contrast, cell density was significantly reduced in the LPS‐treated group, with obvious morphological changes. However, cells in the LPS + LIPUS group showed significant improvement in density and morphology. CCK‐8 assay showed increased OD values in the LPS + LIPUS group, indicating that LIPUS treatment significantly enhanced the survival and proliferation of BV2 cells (Figure 5A). These findings suggest that LIPUS supports cell viability and promotes proliferation.

### 3.5 Quantitative PCR

As shown in Figure 5B, LIPUS intervention significantly increased the expression levels of anti‐inflammatory factors IL‐10 and Arg‐1, while decreasing the expression of pro‐inflammatory factors IL‐6 and TNF‐α in BV2 cells, indicating that LIPUS regulates microglial polarization.

### 3.6 Cellular Immunofluorescence

**FIGURE 6:**
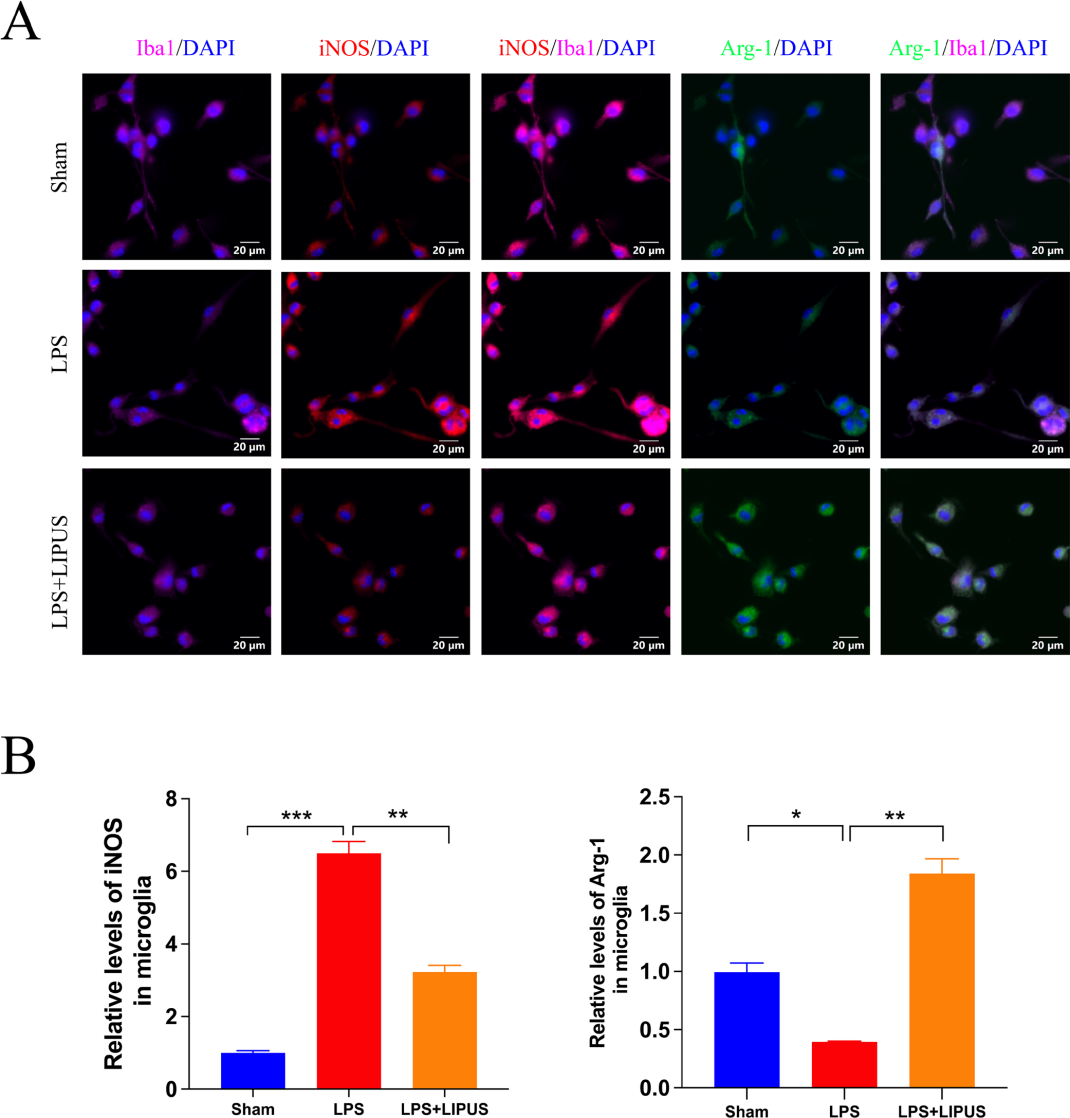
LIPUS inhibited microglia polarization to M1 and promoted microglia polarization to M2 in LPS‐induced microglia. (A) Immunocytofluorescence staining of iNOS, Arg‐l and Ibal, respectively (Scale bar = 20 um). (B) Quantification of iNOS and Arg‐1 immunofluorescence intensity in each group (*n* = 3).

As shown in Figure 6A, LIPUS‐treated BV2 cells showed significant changes in the expression levels of related proteins, including upregulation of arginase‐1 (Arg‐1) and downregulation of inducible nitric oxide synthase (iNOS). Further quantitative analysis (Figure 6B) showed that compared to the LPS group, the relative level of iNOS in microglia was significantly downregulated (*p* < 0.01), while the relative level of Arg‐1 was significantly upregulated (*p* < 0.01) in the LPS + LIPUS group. These changes are associated with microglial polarization toward the M2 phenotype, further confirming that LIPUS promotes the shift of microglia to an anti‐inflammatory phenotype.

### 3.7 HT22 Cell Transwell Co‐Culture

**FIGURE 7:**
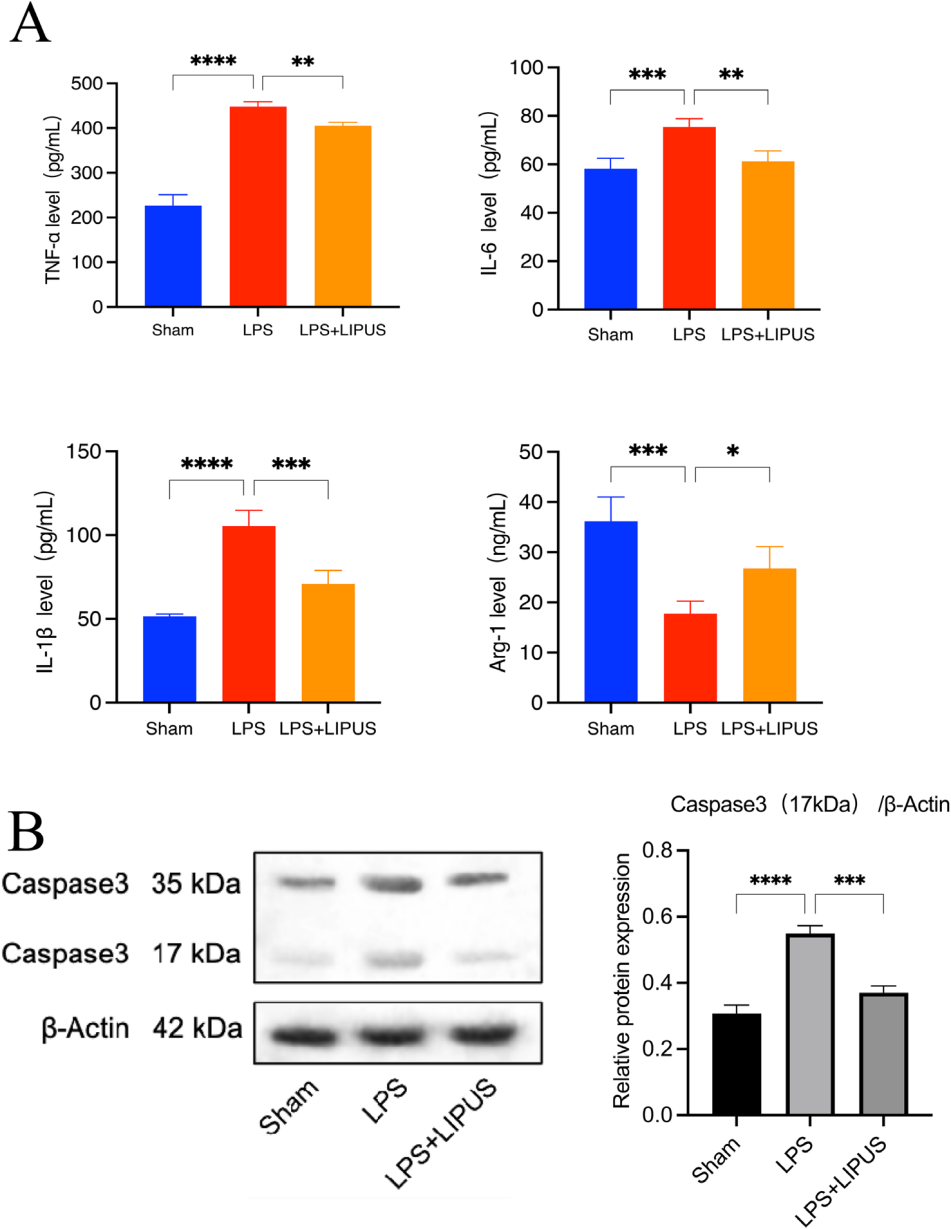
LIPUS inhibits the apoptosis of HT22 neurons by suppressing the expression of inflammatory factors in BV2 cells. (A) Detection of the expression levels of inflammatory factors in the supernatant by ELISA. (B) Detection of Caspase 3 protein level in HT22 neuronal cells by Western Blot (*n* = 3, *p < 0.05, *p < 0.01, **p < 0.001, ***p < 0.0001).

ELISA results (Figure 7A) showed that the expression of pro‐inflammatory factors (TNF‐α, IL‐6, IL‐1β) in supernatants was significantly higher in the LPS group than in the Sham group, while these pro‐inflammatory factors were significantly decreased and the anti‐inflammatory marker Arg‐1 was increased in the LPS + LIPUS group. Western blot detection of Caspase3 protein expression in HT22 neurons in the Transwell co‐culture system (Figure 7B) showed that compared to the Sham group, Caspase3 (17 kDa) expression was significantly increased in the LPS group, while it was significantly decreased in the LPS + LIPUS group compared to the LPS group. These results indicate that in the co‐culture model of BV2 microglia and HT22 neurons, LPS may indirectly activate the endogenous apoptotic pathway in HT22 neurons by regulating inflammatory factors secreted by BV2 cells, while LIPUS may inhibit HT22 neuron apoptosis by suppressing the expression of inflammatory factors in BV2 cells.

### 3.8 Transcriptome Sequencing

After removing reads with adapters, reads with > 5% ambiguous bases (N), and low‐quality reads, the qualified base rate of Q20 was > 90% (Table 2), indicating that sequencing quality met the requirements for transcriptome analysis, allowing subsequent experiments.

**TABLE 2:** Average statistics of sequence quality and alignment information.

Group	SCI	SCI + LIPUS
Valid Ratio (reads)	96.57	97.09
Q20%	97.77	97.83

### 3.9 Bioinformatics Analysis

**FIGURE 8:**
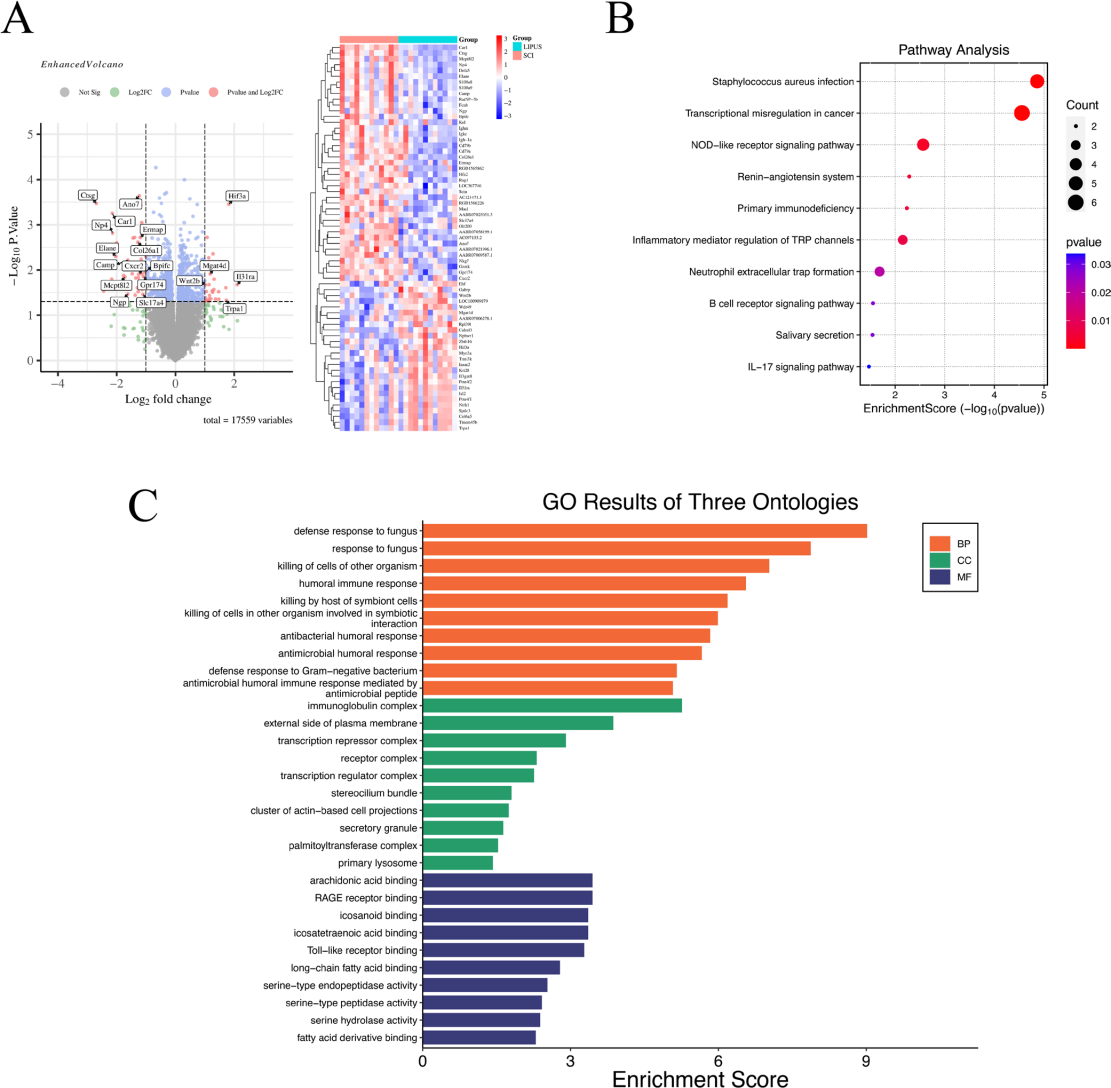
Analysis of Differentially Expressed Genes and Visualization of Functional Enrichment. (A) Differentially expressed genes (B) Kyoto Encyclopedia of Genes and Genomes pathway analysis (C) Gene Ontology enrichment analysis.

Compared to the SCI group, the SCI + LIPUS group had 67 DEGs, including 26 upregulated and 41 downregulated genes (Figure 8A). These genes are mainly involved in immune response, hypoxia adaptation, cell development and differentiation, cell proliferation and apoptosis, and tissue repair.

GO analysis results (Figure 8C) showed that DEGs were mainly enriched in BPs such as “defense response to fungus,” “killing of symbiont cells by host,” “humoral immune response,” “defense response to Gram‐negative bacteria,” and “antimicrobial peptide‐mediated humoral immune response”; CCs including “immunoglobulin complex,” “transcriptional repressor complex,” “receptor complex,” “secretory granule,” “primary lysosome,” “actin‐based cell projection cluster,” and “palmitoyltransferase complex”; MFs such as “arachidonic acid binding,” “RAGE receptor binding,” “Toll‐like receptor binding,” and “fatty acid derivative binding”. KEGG pathway enrichment analysis (Figure 8B) showed that DEGs were mainly enriched in “ *Staphylococcus aureus* infection,” “transcriptional misregulation in cancer,” “NOD‐like receptor signaling pathway,” “primary immunodeficiency,” “TRP channel regulation of inflammatory mediators,” “neutrophil extracellular trap formation,” “B cell receptor signaling pathway,” and “IL‐17 signaling pathway.”

**FIGURE 9:**
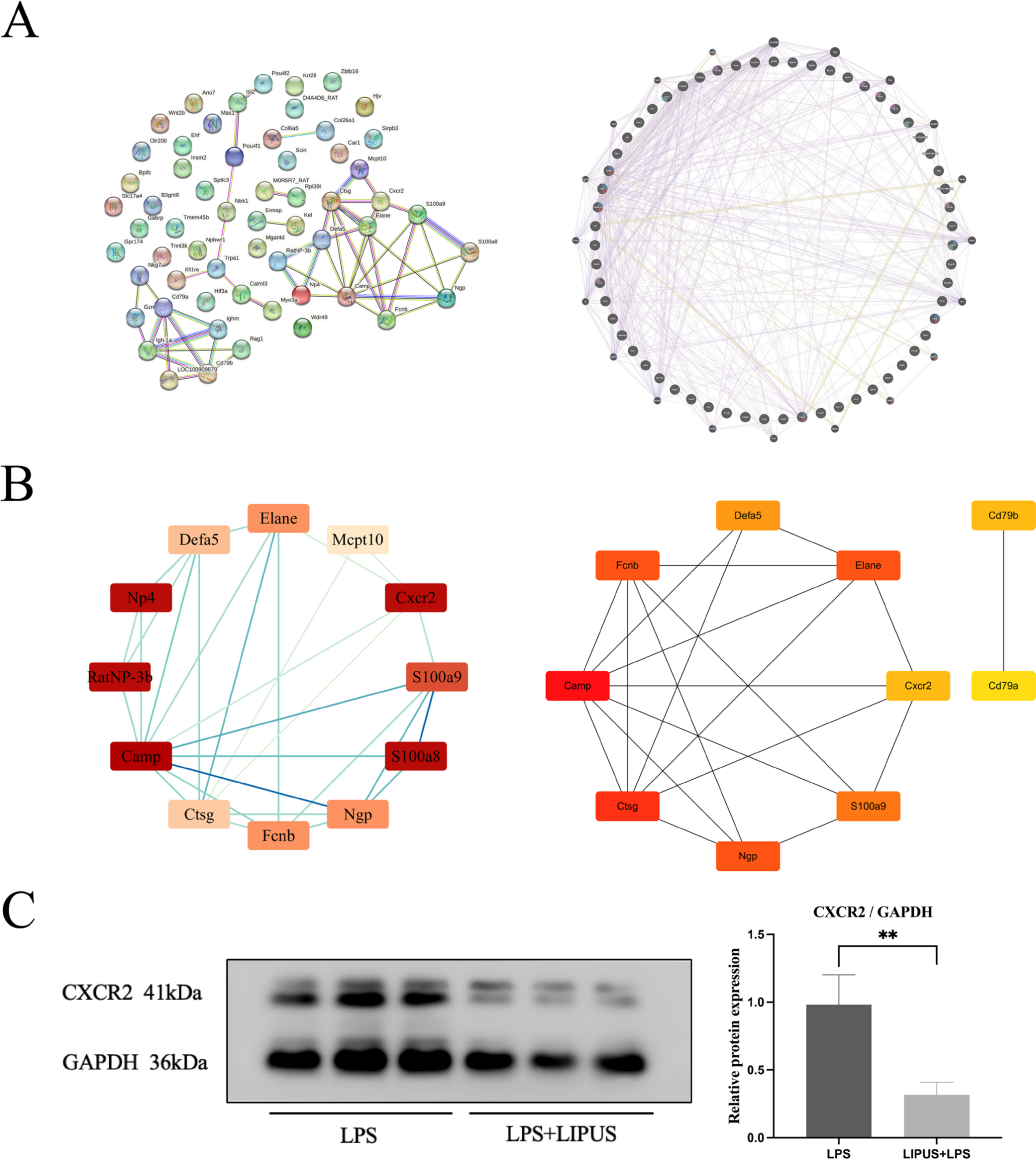
Visualization of Results from Multi‐Algorithm Analysis of Protein–Protein Interaction Networks. (A) STRING protein–protein interaction network (left) and GeneMANIA protein–protein interaction network. Results of the MCODE algorithm (left) and results of the CytoHubba algorithm (right) (C) Detection of CXCR2 protein level in BV2 cells by Western Blot (*n* = 3, **p < 0.01).

The Search Tool for the Retrieval of Interacting Genes (STRING) and Genemania databases were used to analyze the PPI network of DEGs (minimum interaction score > 0.4), which was visualized (Figure 9A). The Cytoscape plugin MCODE was used to identify important modules (parameters: degree cutoff = 2, node score cutoff = 0.2, k‐core = 2, max. depth = 100). A significant module consisting of 12 nodes and 36 edges was identified, involving genes such as Elane, Defa5, Mcpt10, Np4, Cxcr2, RatNP‐3b, S100a9, Camp, S100a8, Ctsg, Ngp, and Fcnb. Further hub gene mining in the PPI network using the MCC algorithm in CytoHubba identified 10 candidate genes, including Defa5, Cd79b, Fcnb, Elane, Camp, Cxcr2, Cd79a, Ctsg, S100a9, and Ngp (Figure 9B). Intersection of results from the two algorithms identified 8 hub DEGs: Elane, Defa5, Cxcr2, S100a9, Camp, Fcnb, Ctsg, and Ngp. These genes are all enriched in innate immune regulation and inflammatory cascade pathways. Among them, Cxcr2 was selected as key target for subsequent experimental validation (Western Blot). As shown in Figure 9C, its downregulation indicates that LIPUS treatment exerts anti‐inflammatory effects and promotes tissue repair by inhibiting microglial activation, reducing neutrophil infiltration and degranulation, and regulating neuroinflammation.

### 3.10 Statistical Analysis Results

All experimental data underwent rigorous statistical analysis to ensure accuracy and reliability. Results showed that LIPUS treatment significantly promoted microglial polarization toward the anti‐inflammatory phenotype and enhanced recovery after SCI in rats (*p* < 0.05). These findings provide strong scientific evidence for the potential application of LIPUS as a therapeutic intervention.

## 4 Discussion

Spinal cord injury is a severe central nervous system injury that can cause significant motor and sensory dysfunction, imposing a heavy burden on patients and society. Current therapeutic strategies for SCI mainly focus on acute injury control [40], surgical intervention, and long‐term rehabilitation training [41]. However, effectively promoting neurological function recovery after injury remains a major challenge. As the primary immune cells in the central nervous system, microglia play complex and critical roles in the pathological process of SCI, participating in cell aggregation, wound compaction, and neural recovery after SCI [42]. The polarization state of microglia (M1 and M2 phenotypes) determines their functional roles after injury [43]. M1 microglia are typically associated with pro‐inflammatory responses and release various inflammatory cytokines such as TNF‐α, IL‐1β, and interferon‐γ, which can limit injury spread in the early stage but may cause further neural damage if overactivated. In contrast, M2 microglia exhibit anti‐inflammatory and repair functions, secreting factors such as IL‐10, transforming growth factor‐β, and brain‐derived neurotrophic factor [44], promoting cell survival, angiogenesis, and neural regeneration. Early activation of M1 microglia is crucial for clearing damaged tissue and preventing infection; however, sustained M1 responses may lead to chronic inflammation and tissue damage. Therefore, regulating the transition from M1 to M2 microglia has become a key focus of SCI therapeutic strategies.

Advances in regenerative medicine have provided new approaches for SCI treatment, including stem cell therapy [45], biological scaffolds [46], tissue engineering [47], and physical therapy. Since its approval by the U.S. Food and Drug Administration for promoting fracture healing in 1994, LIPUS, as a physical therapy, has received increasing attention. LIPUS enhances cell proliferation and differentiation through biological effects of mechanical stress, improves local blood circulation, and regulates inflammatory responses [48]. Its main mechanisms involve pathways such as nuclear factor‐κB (NF‐κB), mitogen‐activated protein kinase (MAPK), and phosphatidylinositol 3‐kinase/protein kinase B (PI3K/Akt) [49]. Although our study highlights the effectiveness of LIPUS as a standalone intervention, its potential synergistic effects when combined with established treatments may yield greater therapeutic benefits. Several studies have reported that LIPUS can be used in combination with various therapies, showing significant clinical potential. For example, bone marrow mesenchymal stem cell (BMSC) transplantation has been shown to improve functional recovery after SCI, and LIPUS has been reported to enhance BMSC viability and neurotrophic factor expression in vitro [49]. Guo et al. reported that LIPUS promotes osteogenic differentiation of mesenchymal stem cells (MSCs), inhibits osteoclast differentiation and gene expression, and activates molecular and cellular responses involved in fracture healing, thereby accelerating callus formation and improving biomechanical properties of healing bone [50]. Ning et al. demonstrated that LIPUS enhances the viability and neurotrophic factor expression of bone marrow MSCs (BMSCs) in vitro, and co‐transplantation of LIPUS‐pretreated BMSCs promotes better functional recovery after SCI [51]. This multifaceted approach not only opens new avenues for SCI repair but also consolidates the role of LIPUS in regenerative medicine.

This study explored the role of LIPUS in promoting post‐SCI recovery. Using BBB scoring and histological analysis, we found that LIPUS treatment significantly improved neurological function recovery and tissue morphological repair in rats after SCI. Immunofluorescence staining results showed that LIPUS significantly reduced the activity and number of microglia after SCI, thereby alleviating inflammation and promoting tissue repair. This transition may be mediated by regulating key signaling pathways, such as inhibiting NF‐κB and activating the PI3K/Akt pathway [52]. Recent studies suggest that LIPUS may exert its effects by activating mechanosensitive ion channels, which can trigger intracellular signaling cascades, promoting cellular metabolism and inflammatory regulation [53]. Additionally, qPCR analysis confirmed the regulatory effect of LIPUS on microglial polarization. Although the exact cellular mechanism of LIPUS remains unclear, CCK‐8 assay showed that LIPUS increased the survival and proliferation of BV2 cells, which may be related to enhanced cellular metabolism and energy production. RNA sequencing and bioinformatics analysis identified 67 DEGs (26 upregulated, 41 downregulated) in the LIPUS group compared to the SCI group. Among upregulated genes, increased expression of Isl2 (related to neuronal development), Il31ra (regulating immune inflammation), Trpa1 (mediating pain signals), and Hif‐3α (improving hypoxia adaptation) plays roles in neuronal regeneration, limiting inflammation scope, alleviating pain, and regulating the local microenvironment, respectively. Among downregulated genes, decreased expression of Ctsg (inhibiting excessive activation of immune cells), Igκc (weakening humoral immune attack), S100a9 (inhibiting M1 polarization of microglia and neuroinflammation), and Cxcr2 (reducing microglial activation and migration) collectively alleviates immune damage. GO analysis showed that DEGs are enriched in processes such as immune defense, inflammatory signaling cascades, extracellular matrix remodeling, and ion transport, involving localization in secretory granules and the outer membrane, and regulating immune responses through mechanisms such as exocytosis and transmembrane signal transduction. KEGG pathway analysis indicated significant enrichment of DEGs in the NOD‐like receptor signaling pathway and IL‐17 signaling pathway: the former activates inflammasomes to drive IL‐1β maturation, while the latter recruits inflammatory cells, collectively forming an immune regulatory network. These findings suggest that LIPUS reduces immune attack in the injury area by regulating these signaling pathways, providing directions for elucidating its molecular mechanisms in treating SCI.

In conclusion, LIPUS exerts positive effects on post‐SCI recovery through multiple mechanisms. It not only regulates inflammatory responses and the immune environment but also promotes cell survival and proliferation. Understanding these mechanisms provides a scientific basis for developing new therapeutic strategies, potentially enabling more effective SCI repair in future clinical applications and opening possibilities for LIPUS use in other neural injuries and inflammatory diseases. Future research will focus on optimizing LIPUS treatment parameters and protocols to achieve optimal therapeutic effects, and exploring its potential combination with other therapies such as stem cell transplantation and biomaterials.

Currently, the clinical management of spinal cord injury (SCI) primarily relies on surgical decompression and pharmacotherapy. Although surgical decompression can relieve physical compression, it involves invasive procedures with inherent risks of infection, hemorrhage, and anesthesia complications, and it often fails to reverse secondary neuronal damage. High‐dose methylprednisolone (MP), considered a standard pharmacological intervention, is frequently associated with severe systemic adverse effects, including gastrointestinal bleeding, susceptibility to infection, and femoral head necrosis. Furthermore, while stem cell transplantation holds promise for neural regeneration, its clinical application is hindered by challenges such as ethical controversies, potential immunogenicity, tumorigenicity, and low cell survival rates in the harsh lesion microenvironment. In contrast, LIPUS offers a distinct therapeutic advantage as a noninvasive physical modality. It avoids the trauma and complications associated with surgery, eliminates the systemic toxicity of high‐dose drugs, and circumvents the biological risks of cell therapy. As a safe, targeted, and “green” therapeutic strategy, LIPUS demonstrates significant potential for clinical translation, particularly in modulating the local immune microenvironment without disturbing systemic homeostasis.

Despite providing positive evidence for the application of LIPUS in SCI recovery, we acknowledge certain limitations of this study. For example, the study primarily focused on the effect of LIPUS on microglial polarization, with insufficient exploration of other potential cell types and molecular mechanisms. Furthermore, the efficacy of LIPUS is highly dependent on acoustic parameters such as intensity, frequency, and duty cycle. In this study, the selection of intensities (30, 90, and 180 mW/cm^2^) was based on a comprehensive consideration of literature evidence, anatomical characteristics, and safety profiles. While 30 mW/cm^2^ served as the FDA‐approved baseline, higher gradients (90 and 180 mW/cm^2^) were applied to compensate for the significant acoustic energy attenuation caused by the anatomical depth of the spinal cord. Although our pilot experiments confirmed the safety of 180 mW/cm^2^, we did not determine the precise “minimum effective dose” or “maximum tolerance dose” using finer gradients. Optimizing these variables based on specific pathological conditions remains a key area for further research. Additionally, the relatively small sample size may affect the generalizability of results, and concerns regarding the long‐term safety of LIPUS are also crucial. Future trials with larger sample sizes are needed to rigorously evaluate the safety and tolerability of LIPUS under different treatment parameters.

## Author Contributions

All authors contributed to the study design, implementation, and data collection. X.L., Y.L., W.L., T.W., H.Z., and Z.D. performed the experiments. X.L. drafted the manuscript. X.L., Y.L., H.S., and H.Z. analyzed the data and interpreted the results. X.Z., X.W., X.L., Y.L., W.L., and T.W. designed the study. All authors contributed to the decision to submit the results for publication.

## Funding

This study was supported by the Youth Science and Technology Talent Project of the Chinese People's Liberation Army (2020QN06125), Shanghai Dawn Program (23SG35), Shanghai Oriental Talents (QNWS2024082), Fundamental Research Special Project of Changhai Hospital (2023PY17), and Changhong Program of Changhai Hospital.

## Disclosure

Transparency, Rigor, and Reproducibility Statement: This study aimed to explore the effect of low‐intensity pulsed ultrasound (LIPUS) on spinal cord injury (SCI) recovery, fully adhering to ethical research standards. All data, methods, and protocols were comprehensively recorded, with results transparently presented in the manuscript. Statistical analyses, experimental procedures, and intervention protocols were described in detail to ensure accurate interpretation and verification of results.

A rigorous experimental design was adopted to ensure result reliability: 88 male Sprague–Dawley rats were used to establish a clear SCI model, with well‐defined grouping criteria (sham operation group, SCI group, and SCI + LIPUS group). Standardized injury procedures and intervention protocols were implemented to minimize variability. Objective assessment tools such as the BBB locomotor rating scale were used for blinded scoring to prevent bias. In vitro experiments used standardized conditions, including validated cell lines (BV2 microglia) and established LIPUS parameters. Appropriate statistical tests, including independent *t*‐tests and ANOVA, were applied, with a clearly defined significance level (*p* < 0.05).

For reproducibility: Animal and cell culture methods, LIPUS parameters, histological and immunofluorescence staining protocols, and statistical analyses were described in detail in the manuscript. All reagents, equipment, and their sources were specified. Results were validated through repeated measurements and triplicates in molecular and cellular assays. Ethical approval and adherence to the *Guide for the Care and Use of Laboratory Animals* of the National Institutes of Health ensure reproducibility in similar ethical contexts.

## Ethics Statement

All animal experiments involved in this study were performed in strict compliance with the Guide for the Care and Use of Laboratory Animals issued by the National Institutes of Health. The experimental protocol was reviewed and formally approved by the Experimental Animal Ethics Committee of Shanghai Changhai Hospital (Approval ID: CHEC(A.E)2025–033). The approval was granted via a formal meeting review (Meeting No.: M(AE)2025–002‐2025‐04‐23) on April 23, 2025.

## Conflicts of Interest

The authors declare no conflicts of interest.

## Data Availability

Data sharing not applicable to this article as no datasets were generated or analysed during the current study.
